# The Use of Biopsy and “No-Biopsy” Approach for Diagnosing Paediatric Coeliac Disease in the Central European Region

**DOI:** 10.1155/2019/9370397

**Published:** 2019-11-15

**Authors:** Petra Riznik, Márta Balogh, Piroska Bódi, Luigina De Leo, Jasmina Dolinsek, Ildikó Guthy, Judit Gyimesi, Ágnes Horváth, Ildikó Kis, Martina Klemenak, Berthold Koletzko, Sibylle Koletzko, Ilma Rita Korponay-Szabó, Tomaz Krencnik, Tarcisio Not, Goran Palcevski, Éva Pollák, Daniele Sblattero, István Tokodi, Matej Vogrincic, Katharina Julia Werkstetter, Jernej Dolinsek

**Affiliations:** ^1^University Medical Centre Maribor, Department of Paediatrics, Gastroenterology, Hepatology and Nutrition Unit, Maribor, Slovenia; ^2^Markusovszky Teaching Hospital, Szombathely, Hungary; ^3^Pándy Kálmán Hospital, Gyula, Hungary; ^4^IRCCS Burlo Garofolo Trieste, Institute for Maternal and Child Health, Trieste, Italy; ^5^Municipality of Maribor, Project Office, Maribor, Slovenia; ^6^Jósa András County Hospital, Nyíregyháza, Hungary; ^7^Heim Pál National Paediatric Institute, Coeliac Disease Centre, Budapest, Hungary; ^8^Csolnoky Ferenc County Hospital, Veszprém, Hungary; ^9^St. Barbara County Hospital, Tatabánya, Hungary; ^10^Stiftung Kindergesundheit (Child Health Foundation) at Dr. von Hauner Children's Hospital, LMU Munich, Munich, Germany; ^11^Dr. von Hauner Children's Hospital, Clinical Medical Centre, LMU Munich, Munich, Germany; ^12^Department of Pediatrics, Gastroenterology and Nutrition, School of Medicine Collegium Medicum University of Warmia and Mazury, Olsztyn, Poland; ^13^University of Debrecen, Faculty of Medicine, Department of Paediatrics, Debrecen, Hungary; ^14^University Hospital Rijeka, Department for Gastroenterology, Paediatric Clinic, Rijeka, Croatia; ^15^Ajka County Hospital, Ajka, Hungary; ^16^University of Trieste, Trieste, Italy; ^17^St. George Fejér County University Teaching Hospital, Székesfehérvár, Hungary; ^18^University Medical Centre Maribor, Department of Informatics, Maribor, Slovenia; ^19^Medical Faculty, Department of Paediatrics, University of Maribor, Maribor, Slovenia

## Abstract

**Objectives:**

The current European Society for Paediatric Gastroenterology, Hepatology, and Nutrition (ESPGHAN) guidelines introduced the option to diagnose coeliac disease (CD) in children and adolescents without upper endoscopy if the defined criteria are met. The aim of our study was to evaluate how frequently paediatric gastroenterologists in Central Europe used the “no-biopsy” approach and how often the duodenal biopsy could have been omitted.

**Methods:**

Medical records of patients aged < 19 years diagnosed with CD in 2016 from five European countries were analysed, focusing on levels of transglutaminase antibodies (TGA) at the time of diagnosis and on whether the diagnosis was confirmed using duodenal biopsy or “no-biopsy” approach. Clinical presentation and delays until final diagnosis were analysed according to diagnostic approach.

**Results:**

Data from 653 children (63.9% female, median age: 7 years, range: 7 months-18.5 years) from Croatia, Hungary, Germany, Italy, and Slovenia were analysed. One fifth (*n* = 134) of included children were asymptomatic at diagnosis. Of 519 symptomatic children, 107 (20.6%) were diagnosed by the “no-biopsy” approach. Out of the remaining 412 children who underwent duodenal biopsies, 214 (51.9%) had TGA ≥ 10 times upper level of normal (ULN) and would have been eligible for the “no-biopsy” approach. Signs and symptoms of malabsorption were more frequent in children diagnosed without duodenal biopsies. There were no differences in diagnostic delays with respect to the diagnostic approach.

**Conclusion:**

In this cohort, about 60% of symptomatic CD patients could have been diagnosed without duodenal biopsies. The aim of the “no-biopsy” approach was to make the diagnostic procedure less challenging without compromising its reliability. However, this option was applied only in 20%, in spite of fewer burdens to the family and reduced costs. The reasons for this discrepancy are unknown. Physicians should be made more aware about the reliability of CD diagnosis without biopsies when the ESPGHAN guidelines for CD diagnosis are followed.

## 1. Introduction

Coeliac disease (CD) is a lifelong systemic autoimmune disorder, elicited by gluten and related prolamins in genetically susceptible individuals. Traditionally defined as gluten-related enteropathy, it is one of the most common chronic illnesses with very diverse clinical presentation, involving intestinal and extraintestinal manifestations [1]. Histological findings of villous atrophy and crypt hyperplasia with increased levels of intraepithelial T lymphocytes from duodenal biopsies, classified according to the Marsh-Oberhuber, have been regarded as the gold standard for diagnosing CD [2–4].

The first diagnostic criteria for CD were the Interlaken criteria, formalised in 1969 by the experts in the newly born European Society for Paediatric Gastroenterology, today known as ESPGHAN (European Society for Paediatric Gastroenterology, Hepatology, and Nutrition). Three duodenal biopsies (initial on gluten, after treatment with a gluten-free diet, and after gluten challenge) were required for the confirmation of the diagnosis, and these criteria served worldwide as the accepted diagnostic standard for over 20 years [5]. In the revised ESPGHAN criteria, published in 1990, the need for gluten challenge for children over the age of 2 years was removed and serological tests were added to the diagnostic procedure [6, 7]. One duodenal biopsy was required for the confirmation of the diagnosis and with clinical and serological improvement after introduction of gluten-free diet; no further biopsies were needed [6].

In the current ESPGHAN guidelines, published in 2012, the initial diagnostic step is the determination of CD-specific IgA autoantibodies against type-2 (tissue) transglutaminase (TGA) together with total IgA in serum [1]. In case of low or undetectable total IgA, an IgG-based test should be used. Positive autoantibodies imply a high probability of mucosal atrophy, and to confirm the diagnosis, an upper endoscopy with multiple duodenal biopsies should be performed [1]. However, these guidelines are the first allowing paediatric gastroenterologists to diagnose the disease without intestinal biopsy if all of the following criteria are fulfilled: the child shows symptoms and signs suggestive of CD, has high levels of TGA antibodies above 10 times upper level of normal (ULN), a positive confirmatory EMA test in a 2nd blood sample, specific HLA DQ2 or DQ8 genes, and consent of the patient and caregiver for this “no-biopsy” diagnostic approach [1]. A year later, the so-called “no-biopsy” approach, proposed by ESPGHAN, was adopted by the British Society for Paediatric Gastroenterology, Hepatology and Nutrition (BSPGHAN) [8]. The only difference between the two guidelines is that the joint BSPGHAN and Coeliac UK guidelines allow the substitution of 2nd EMA test with 2nd strongly positive TGA test, where EMA test is not locally available. However, the serum of the patient should be saved for later EMA testing [8]. On the other hand, the guidelines by the North American Society for Paediatric Gastroenterology, Hepatology, and Nutrition (NASPGHAN) recommend the intestinal biopsy for the confirmation of the diagnosis of CD in all cases, regardless of the value of TGA [9, 10].

Although the so-called “no-biopsy” approach could have been used for the past 6 years, to our knowledge, there is not much data on how often the diagnosis was confirmed without duodenal biopsy.

The aim of our study was to evaluate how frequently the “no-biopsy” approach was used to diagnose children with CD in Central Europe (CE) and how often the duodenal biopsy could have been omitted.

## 2. Materials and Methods

The study, conducted in the scope of the Focus IN CD project (CE 111) and co-financed by the Interreg CE Programme, was carried out between the end of March and the middle of August 2017. Twelve partners from five CE countries (Croatia, Germany, Hungary, Italy, and Slovenia) participate in the project. Paediatric gastroenterologists from the participating regions were asked by the regional project partners to complete a web-based survey, providing anonymized medical records of children and adolescents below 19 years of age who were diagnosed with CD in 2016. In Croatia, Hungary, and Slovenia, the majority of CD patients diagnosed by paediatric gastroenterologists during this year were included. The questionnaire (https://www.interreg-central.eu/Content.Node/surveys.html) was translated into the languages of all project partners and focused on clinical presentation, diagnostic methods used, and management of CD. We analysed medical records of all included CD patients, focusing on levels of TGA at the time of diagnosis and on whether the diagnosis was confirmed using duodenal biopsy showing Marsh 2-3 lesion or “no-biopsy” approach. We also compared diagnostic approach with clinical presentation of the disease (with or without signs and symptoms of malabsorption) and the diagnostic delays, calculated as the duration from the first symptoms to the confirmation of the diagnosis. Statistical analysis was performed using IBM SPSS Statistics 22.0 for Windows. One-way ANOVA, chi-square test, and Kruskal-Wallis *H* test with post hoc test were used for the analysis.

The study was approved by the National Medical Ethics Committee of the Republic of Slovenia (0120-383).

## 3. Results

**Figure 1:**
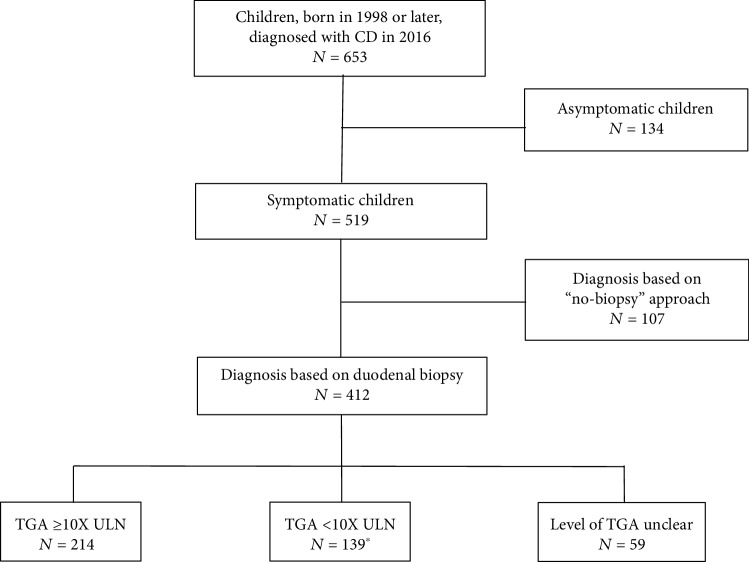
Diagnostic approach in children with CD in CE. ^∗^15 patients had IgA deficiency.

Data from 653 children and adolescents from Croatia (*n* = 66), Germany (*n* = 69), Hungary (*n* = 382), Italy (*n* = 82), and Slovenia (*n* = 54) were available for the analysis. Median age of the children at the time of diagnosis was 7 years (range: 7 months-18.5 years), 63.9% were female. One fifth (*n* = 134) of included children were asymptomatic at the confirmation of the diagnosis (65.7% had TGA ≥ 10× ULN). Analysis of the diagnostic procedure (Figure 1) showed that 20.6% (*n* = 107) of symptomatic children were diagnosed using “no-biopsy” approach. Out of 412 children who underwent duodenal biopsy, 51.9% (*n* = 214) had TGA ≥ 10× ULN and could be considered as eligible for the “no-biopsy” approach (Table 1). Final diagnosis in this case should have been confirmed by positive genetic tests and positive EMA in the 2nd blood sample. However, since duodenal biopsy was chosen as confirmatory test, confirmatory serology and genetic tests were often not performed.

Of 519 symptomatic children, endoscopy with biopsies to confirm CD was performed in 412 (79.4%). Proportion of patients diagnosed using biopsy approach was highest in Croatia (93.1%) and was significantly higher compared to Slovenia (62.8%) (*p* < 0.05). No statistically significant differences between other countries were found.

Clinical presentation of patients diagnosed with or without biopsy was analysed separately (Table 2).

We also compared diagnostic delays between children diagnosed without biopsy and those who underwent duodenal biopsy. In order to be able to calculate diagnostic delays, we excluded symptomatic patients with unclear data about the time of the first symptoms or first visit to the paediatric gastroenterologist (*n* = 126). Data from 393 children were available for the analysis.

There were no differences between children diagnosed without duodenal biopsy and those diagnosed with biopsy who would have been eligible for the “no-biopsy” approach. Interval from the first visit at the paediatric gastroenterologist to the confirmation of the diagnosis was longer in the “no-biopsy” group compared to the group of children who underwent biopsy and were not eligible for the “no-biopsy” approach (*p* < 0.05), without differences in total delay (from symptoms to final diagnosis) (Table 3).

## 4. Discussion

The ESPGHAN guidelines for the diagnosis of CD for the last 6 years allow the possibility of using “no-biopsy” approach in children and adolescents if certain criteria are fulfilled. This diagnostic approach had been shown to be safe with a positive predictive value for enteropathy of >99% in a large prospective international study including 707 paediatric patients [11]. Our data provided by paediatric gastroenterologist for patients diagnosed with CD in 2016 shows that only about 20% of children in Central Europe were diagnosed without duodenal biopsy, although about 60% would have been eligible based on the level of TGA being higher than 10 times ULN. The highest proportion of children diagnosed with the “no-biopsy” approach was reported from Slovenia, where CD is diagnosed in only few centres and where several awareness-rising campaigns have been carried out during the last few years. However, further half of the CD children diagnosed with duodenal biopsy would also have been eligible for the “no-biopsy” approach since their TGA levels were very high (≥10× ULN). In the majority of these patients, genetic tests and confirmatory EMA were not performed, since duodenal biopsy was chosen as a confirmatory test. No information on how many of them had perhaps been additionally tested for EMA in a second sample was available, since this was not specifically asked for in patients who underwent duodenal biopsy. Altogether, at least 60% of children diagnosed in Central Europe could have been considered to be diagnosed without duodenal biopsy. In a substantial number of patients (14%), we were not able to define the eligibility for the “no-biopsy” approach because of the incomplete data on either TGA levels or the cut-off values of the used tests. This might be one of the reasons for uneven proportion of very high levels of TGA among countries, especially in Croatia, were the percentage of TGA ≥ 10 times ULN was the lowest.

The majority of all patients presented with at least one sign or symptom of malabsorption, with a significantly higher proportion in patients, who were diagnosed using “no-biopsy” approach compared to those who were not eligible (TGA levels < 10 times ULN) for the “no-biopsy” approach.

Our data show that duodenal biopsy is still performed in majority of children with CD, regardless of the possibility of the “no-biopsy” approach. The reasons for this might be a higher trust in biopsy results compared to serology, possibly because the physicians want to avoid misdiagnosis of this lifelong disease where compliance with the diet is extremely important. Another possible reason for choosing biopsy pathway in children can be the existing belief that genetic tests and serological tests are expensive compared to duodenal biopsy where biopsy is made with existing equipment and existing personnel. Also, there could be a perception that using the “no-biopsy” approach would be more time consuming, since after an endoscopy, the child can be put on a diet immediately without a fear of influencing further tests that need to be done if a “no-biopsy” approach is chosen. It is also important to note that in many centres, endoscopy is readily available, and serological tests are performed outside the institution. This creates an impression that endoscopy is more accessible and is associated with lower risk of long diagnostic delays and false diagnosis. However, based on our study, no difference in diagnostic delays was found with the respect to different diagnostic procedure used.

Our results are similar to the study of Bishop et al. [12], where more than half of the included patients fulfilled ESPGHAN criteria for the “no-biopsy” approach but the guidelines were not adopted since different laboratory testing platforms that were used have not been sufficiently validated to completely trust the serological results [12]. Also, NASPGHAN guidelines for diagnosing CD do not include the possibility of “no-biopsy” approach since there is no standardisation of serological tests in the USA [10]. On the other hand, European studies clearly confirmed the reliability of serological tests in the “no-biopsy” approach [11, 13]. In the study of Werkstetter et al. [11], the current ESPGHAN guidelines regarding the “no-biopsy” approach were prospectively evaluated and it was confirmed that children could be safely diagnosed without biopsy, based on the reliable serological kits [11]. Moreover, recent prospective validation studies show that for the “no-biopsy” approach, HLA analysis is probably not necessary [11, 13].

Another possible reason for choosing duodenal biopsy in CD diagnosis is the potential risk of missing other diseases, which would have been detected if upper endoscopy was performed [14]; however, this has not been demonstrated in studies on children and adolescents with suspected CD [11, 15].

One of the concerns related to the “no-biopsy” approach might also be lack of implementation of existing ESPGHAN guidelines for this approach in general practice [14]. The awareness about CD and about existing guidelines is low among healthcare professionals [16–19] and this can rise a suspicion that proposed diagnostic standards brought by the guidelines are not fully met, leading to uncertain diagnosis, with either over- or underdiagnosis of CD [14].

When choosing to perform a duodenal biopsy, several pitfalls in the interpretation of duodenal biopsies regardless of them being a gold standard in diagnosing CD must be considered. Histological analysis has been reported to lack diagnostic accuracy owing to the high interobserver variability, differences between routine and more specialised pathology laboratories, low rates of correct orientation of biopsy samples, and low number of samples taken. These factors can lead to inadequate interpretation of mucosal changes [14, 20]. Adequate sampling by the endoscopist aware of the potential for patchy nature of the enteropathy includes at least four biopsies from the duodenum distal to the papilla of Vateri and at least one from the duodenal bulb during a gluten-containing diet [1, 4, 14, 20].

The major advantage of a “no-biopsy” approach in children and adolescents is the avoidance of upper endoscopy, which, in many centres, requires general anaesthesia or deep sedation, leading to higher costs in comparison to serological diagnosis [14]. Risk of multiple duodenal biopsies and risks of general anaesthesia or deep sedation and endoscopy itself must also be considered. Patients and parents/caregivers must be informed if they are eligible for the “no-biopsy” approach. They should be aware of the potential benefits and disadvantages of two different diagnostic pathways before they decide to undergo duodenal biopsy.

One of the limitations of our study is the retrospective nature of assessment of existing healthcare records and the uneven number of included patients between participating countries, with more patients in Hungary than in other countries. One possible limitation is also the use of different serological tests to determine the levels of TGA among countries, which might partly explain the different proportion of TGA ≥ 10 times ULN among countries [11]. Since we have not anticipated that “no-biopsy” approach is used so rarely, we had not included any question on the possible reasons for performing the biopsy in cases where it might not be needed. Therefore, our results can serve as a basis for further studies of this issue.

## 5. Conclusion

It has been shown that high levels of TGA accurately predict advanced histological changes of the type Marsh 2-3 in the duodenum, and no concern in misdiagnosing CD is justified. In our study, 60% of patients were eligible for the “no-biopsy” approach. Nevertheless, it is important that the diagnosis is confirmed in specialised gastroenterology services with standardised serological tests and not in the general practice.

The aim of the “no-biopsy” approach proposed by current ESPGHAN guidelines is to make the diagnostic procedure less challenging without compromising its reliability. It is therefore important to raise the awareness about CD and possible diagnostic approaches among physicians in order to increase compliance to the guidelines with respect to the “no-biopsy” approach.

## Figures and Tables

**Table 1:** Data on serological testing in symptomatic patients diagnosed with CD who underwent duodenal biopsies in Central Europe.

	Croatia (*N* = 58)	Germany (*N* = 53)	Hungary (*N* = 302)	Italy (*N* = 61)	Slovenia (*N* = 45)	Central Europe (*N* = 519)
“No-biopsy” approach, *n* (% of all patients)	4 (6.9%)	12 (22.6%)	64 (21.2%)	10 (16.4%)	17 (37.8%)	**107 (20.6%)**
Duodenal biopsy, *n* (% of all patients)	54^**∏**^ (93.1%)	41 (77.4%)	238 (78.8%)	51 (83.6%)	28^∏^ (62.2%)	**412 (79.4%)**
TGA ≥ 10 times ULN						
Yes (% within a group)	16 (29.6%)	21 (51.2%)	139 (58.4%)	27 (52.9%)	11 (39.3%)	**214 (51.9%)**
No (% within a group)	23 (42.6%)	8 (19.5%)	72 (30.3%)	21 (41.2%)	15 (53.6%)	**139 (33.7%)**
Unclear (% within a group)	15 (27.8%)	12 (29.3%)	27 (11.3%)	3 (5.9%)	2 (7.1%)	**59 (14.3%)**

^∏^ *p* < 0.05.

**Table 2:** Clinical presentation (with or without symptoms and signs of malabsorption) and diagnostic approach of children with CD. In the group of patients who underwent duodenal biopsy, signs and symptoms of malabsorption were slightly more common in those who would have been eligible for the “no-biopsy” approach (67.8% vs 59.6%; NS). There were no significant differences in clinical presentation between children, diagnosed using “no-biopsy” approach and those who underwent duodenal biopsy but would have been eligible (by the TGA level ≥ 10× ULN) for the “no-biopsy” approach (72.0% vs 67.8%; NS). However, signs and symptoms of malabsorption were significantly more common in patients who were diagnosed using “no-biopsy” approach in comparison to those that were not eligible for the “no-biopsy” approach (72.0% vs 59.6%; *p* < 0.05).

	“No-biopsy” approach	Duodenal biopsy	
		Eligible^∗^ for “no-biopsy”	Not eligible for “no-biopsy”
Malabsorptive (% within group)	77^#^ (72.0%)	145 (67.8%)	118^#^ (59.6%)
Non-malabsorptive (% within group)	30 (28.0%)	69 (32.2%)	80 (40.4%)
Number of patients	**107**	**214**	**198**

^∗^Eligible by *TGA* *level* ≥ 10× ULN. ^#^*p* < 0.05 “no-biopsy” vs not eligible for the “no-biopsy” group.

**Table 3:** Diagnostic delays in children with CD with the respect to the diagnostic procedure.

	“No-biopsy” approach (*N* = 78)	Duodenal biopsy	
		Eligible^∗∗^ for “no-biopsy” approach (*N* = 163)	Not eligible for “no-biopsy” approach (*N* = 152)
Time from 1st symptom until 1st visit to PaedGI, median (Q1; Q3)	4.5 m (2 m; 9.5 m)	5 m (2 m; 11 m)	5 m (2 m; 12 m)
Time from 1st visit to PaedGI until diagnosis, median (Q1; Q3)	1 m^**#**^ (0 m; 2 m)	1 m (0 m; 2 m)	1 m^**#**^ (0 m; 3 m)
Time from symptoms to diagnosis (diagnostic delay), median (Q1; Q3)	6 m (3 m; 12 m)	6 m (3 m; 12 m)	7 m (4 m; 17 m)

^∗^PaedGI: paediatric gastroenterologist; m: month. ^∗∗^Eligible by TGA level ≥ 10× ULN. ^#^*p* < 0.05 not eligible for “no-biopsy” vs “no-biopsy”.

## Data Availability

The data used to support the findings of this study are available from the corresponding author upon request.
